# Medlar—A Comprehensive and Integrative Review

**DOI:** 10.3390/plants10112344

**Published:** 2021-10-29

**Authors:** Catalina Voaides, Nicoleta Radu, Elena Birza, Narcisa Babeanu

**Affiliations:** 1 Faculty of Biotechnologies, University of Agronomic Sciences and Veterinary Medicine of Bucharest, 59 Marasti Blvd., 011464 Bucharest, Romania; catalina.voaides@biotehnologii.usamv.ro (C.V.); nicoleta.radu@biotehnologii.usamv.ro (N.R.); narcisa.babeanu@biotehnologii.usamv.ro (N.B.); 2 National Institute for R&D in Chemistry and Petrochemistry of Bucharest, 202 Splaiul Independentei, 60021 Bucharest, Romania

**Keywords:** medlar, chemical composition, biological activity

## Abstract

Among fruit plants belonging to the Rosaceae family, medlar (*Mespilus*) can be classified as neglected or underutilized. It is a genus of two species of flowering plants: *Mespilus germanica* (common medlar) and *Mespilus canescens*. Appreciated for its specific taste and flavor, medlar also possesses biological properties (antioxidant and antimicrobial). Despite the special properties of medlar, there are few research papers on this subject. This review paper includes data not only on medlar fruits but also its leaves, bark, and bud flowers. The main identified components are presented, as well as several biological properties, morphological properties, ethnopharmacological uses, and molecular biology analyses emerging from the scientific papers published in this area.

## 1. Introduction

Over thousands of years, rosaceous plants from the temperate areas of the northern hemisphere have played an important role. The appreciated fruits of the *Rosaceae* family (e.g., apples, pears, cherries, apricots, peaches, nectarines, plums, quinces, etc.) are still an important part of the human diet. This plant family, which comprises over 100 genera and 3000 species, has been the third most economically important plant family in temperate regions in last decade [1]. The nutritional and sensory qualities of the edible rosaceous crops are well known. Moreover, the fruits of this family are extremely rich in compounds with strong antioxidant activities (e.g., L-ascorbic acid, phenolics, and flavonoids) and other phytochemicals with important effects on health. The obvious growing interest in almost “forgotten” fruit species as a source of important compounds and their pharmacological, antimicrobial, and gastronomic properties is due in part to the actual problem (of humanity) of the lack of food resources [2].

Among fruit-plants that belong to *Rosaceae* family, medlar (*Mespilus*) can be classified as neglected or underutilized [1]. It is a genus of two species of flowering plants in the subfamily *Maloideae*: *Mespilus germanica* L. (common medlar) and *Mespilus canescens J.B.Phipps.* The first one is a well-known native of Southwest Asia and also Southeastern Europe, while the second species was recently discovered in 1990 in North America [3,4]. The genus *Eriobotrya* (*Eriobotrya japonica*—loquats) is also related and sometimes called the “Japanese Medlar” [3,4].

Despite its Latin name, which means German or Germanic medlar [4], about 3000 years ago *Mespilus germanica* might have been cultivated in the Caspian Sea region of Northern Iran and the Black Sea coasts of modern Turkey [5]. It was introduced to Greece around 700 BC and to Rome at around 200 BC. It seems to have been an important fruit plant during Roman and medieval times. By the 17th and 18th century, however, it was forgotten (when more convenient late-ripening fruits became abundant), but it has begun to be cultivated again today. There are several cultivars grown for their fruit’s quality that include “Hollandia,” “Nottingham,” “Russian” [4], “Dutch” (with the largest fruits, also called “Giant” or “Monstrous”) [4], “Royal,” “Breda giant,” and “Large Russian” [4]. In addition to these cultivars, different varieties/genotypes have been analyzed. Medlar (*Mespilus germanica* L.) has different names in different countries (Table 1).

Several scientific papers analyzed the rediscovered medlar from various points of view: pomological [6,17,18,19,20,21], phenotypic [3,22,23,24,25], phylogenetic [26,27,28,29,30,31,32,33], chemical composition, including polyphenols, flavonoids, other antioxidants compounds, macroelements and microelements [9,17,34,35,36,37,38,39,40,41,42,43,44,45,46,47,48,49], antimicrobial effects [10,50,51,52], the influence of ripening stage [1,17,34,35,36,38,49,53], medicinal benefits [4,24,54,55,56,57], etc.

As previously mentioned, medlar represents one of the neglected fruit plants. No production information regarding this valuable plant worldwide was found, not even in the countries of origin. In Russia, medlar is not cultivated in large quantities and can be found only in botanical and private gardens [2]; in Montenegro, there are no statistics, but the cultivation of this fruit is very low [6]. On the other hand, in Turkey, medlar production is approximately 4134 tons, with 51 tons alone in Tokat province [32]. “Royal,” “Nottingham,” and “Dutch” varieties are grown in commercially producing countries such as Germany and the Netherlands [32], and “Istanbul,” “Italian,” and “Akcakoca 77” varieties are grown in Turkey [32]. In Romania, the medlar has been found since ancient times in the countryside, but it has not been cultivated [58]. There are several commercial varieties on the European and U.S.A. markets: “Nottingham” with aromatic fruits, “Dutch” with fruits larger than average, “Regal” (“Royal”), “Marele Rus” (“Large Russian”), etc. [59]. Today, it is found mainly in Tulcea, Muntenia, and Oltenia regions of Romania [60]. In the United States, medlar trees are mostly grown in private gardens, but several farmers or companies in the northeast recently planted it in small orchards [30].

The present review paper aims to present an almost complete image of the identified chemical compounds in different varieties/genotypes of the *Mespilus germanica* L., as well as their potential biological activities (antioxidant, antimicrobial, and pharmacological) and ethnopharmacological relevance, from scientific papers published in the past two decades. The selection of the articles included in the present review was performed by using the well known electronic databases (Web of Science, Scopus, ScienceDirect, EBSCO, PubMed, and Google Scholar), using specific keywords (“genetic identification,” “chemical composition,” “therapeutic,” “uses,” “anti*” (returning results for “antimicrobial”), “antifungal,” etc.). The validation of the articles was performed by reading each article. In the present review, only articles with significant contributions to this field of research were considered.

## 2. Morphological Analysis

Medlars are hard to grow from seed (germination to seedling requires about 2 years), so most commercial varieties are grafted onto other root stock species in order to improve their performance in different soils, areas, and climates. The best results are achieved by grafting on generative rootstock of medlar (*Mespilus germanica* L.), sorb apple (*Sorbus domestica* L.), a whitethorn (*Crataegus sp*.), wild pear (*Pyrus communis* L.), and vegetative rootstock of pear and quince (Quince A, Quince C, and Ba 29) [4,61]. They are self-fertile; thus, they do not need another tree/plant for pollination: they will produce fruit by the second year [61].

**Figure 1:**
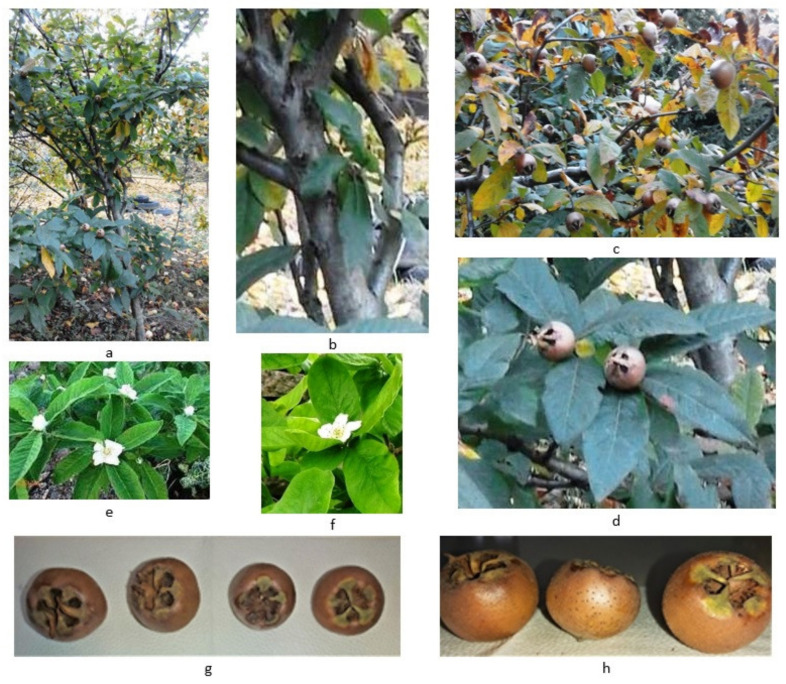
Medlar (**a**)—tree (original); (**b**)–bark (original); (**c**,**d,g,h**)—fruits (original); (**d**) leaves (original); (**e**,**f**)—flowers (original).

In general, all medlar cultivars/genotypes analyzed in the present paper share some common characteristics. They are in a wild form or are commercial cultivars, are slow growing, and are large deciduous spiny shrubs or small trees growing up to 8 m tall (Figure 1a,b).

The fruits are pomes and range from brown (when mature-ripe) to dark brown (overripe), with wide-spreading persistent sepals giving a “hollowed out” aspect to the fruit [4]; sometimes, the fruits are reddish coloured and pear-shaped or apple-shaped, with a diameter between 1.5 and 3 cm and weighing from 10 g to over 80 g (very small fruits—large fruits) [45] (Figure 1c,d,g,h). *M. germanica* fruits are very hard and acidic. The fruits become edible in the winter (among the few that do this) after being softened (“bletted”) by frost or stored naturally for a long period of time. Starting with the softening phase, the skin rapidly acquires a wrinkled texture and turns dark color (chocolate brown); the inside of the fruit is transformed to the consistency and light-flavor of apple sauce. The flavor is described as rich, cidery, and wine-like, dried apples-like or quinces-like [20]. The cultivated plants have larger and sweeter fruits compared to the wild forms [38].

Medlar trees require warm summers and mild winters and prefer sunny, dry locations with slightly acidic soil [4], but Gulcin et al. [45] considered that medlar grows poorly in frost-free areas and on rocks and in poor soils. It is well known that both biological factors (species/cultivar/genotype, age, or pests) and abiotic factors (weather, soil properties, irrigation, planting distance, etc.) have a significant influence on plant tree [18,23]. Thus, the phenological stages are closely related to morphological changes, and the characteristics of fruit trees, as an interannual variability, have been observed [18].

The foliage surface of trees is also influenced by the same factors mentioned above, but, in turn, it influences principal plant processes such as photosynthesis, transpiration, and absorption [23]. Moreover, leaf characteristics (dimensions and shape) can vary significantly between different genotypes within the same species [23], having an important role on plant growth and productivity. The leaves are elongated, lanceolate to obovate (like that of apple), entire or serrulate, dark green, 6–15 cm long, and 3–6 cm wide, and the leaves turn a special red when they acquire senescence (Figure 1d) [21,45] (Table 2). The plant has beautiful white-pink and hermaphrodite flowers in late spring [4] (Figure 1e,f). Flower buds are formed during May–June, and each bud has one flower. With a lifespan between 30 and 50 years, it is considered that *M. germanica* has a fairly short lifetime. [4]. However, there are 100 years old trees in UK [6].

Along with the rediscovery of *Mespilus germanica* L. plants, the medlar fruit has earned its place in human diet by its value. Thus, the fruit is a climacteric one, harvested in October and November and stored (in cold, dark, and aerated conditions, optionally in straw) until it becomes edible in the winter; the complex ripening process is genetically determined [4,39,45]. The green and hard flesh of the fruit softens and changes its color to light brown [53]. The result of this process includes major changes in texture, color, flavor, and aroma [39], resulting in brown (the pulp darkens), softened, and sweeter fruit. The inconveniences of this process include decreasing shelf-life and loss of marketable value [1,53]. Fruit shape may vary and generally include sub-globose or pyriform fruits crowned by foliaceous sepals [1,6]. The medlar shows better pest and climate resistance than most other fruit species of landscaping importance (apples, pears, apricots, peaches, cherries, etc.) [6]. The main characteristics of medlar fruits from research articles are listed in Table 3.

Several authors concluded that changes in structure, texture, color, aroma, and flavor of fruits are directly related to the stage of ripening process (usually presented as Days after full bloom = DAFB) [1,34,35,44]. Thus, at the final stage of the ripening process (207, 174, 187, and 206 DAFB), it was observed that the skin was completely brown, the pulp was whitish (50–60%)–brownish (40–50%) [34,44] or completely dark [1,35], and the fruit soft. The differences in the number of DAFB result from the starting date of the accounting days (10 May, 10 June, 8 May, and 10 May, respectively) [1,34,35,44]. Only Sulusoglu-Durul and Unver [53] did not use the same measure for the ripening stage. However, 25 days after harvest, they observed the same changes as other authors, meaning darkening, softening, dehydration, and flavor development of the fruits. Moreover, out of all the research papers, Sulusoglu-Durul and Unver [53] are the only ones that mentioned tree productivity, which ranged between 5.9 and 17.8 kg (province of Kocaeli, in Northwestern Turkey).

As a result, from various research articles, the main morphometric characteristics of *Mespilus germanica* L. plant parts (fruits and stone) are presented in Table 4. The data indicate a high degree of fruit variability. The main difference between the genotypes is related to their average weight that ranged between 2.9± 0.1 g (at 39 DAFB, unripe stage) [36] and 40,80 g. Several authors [32,41,53] observed that even if there were important differences in fruit weight, diameter, and length (all these parameters being influenced by the genotype), there were no important differences by different years in measurement. Sulusoglu-Durul [53] observed that the fruit weight varied from 9.69 to 24.45 g and the seed numbers ranged from 1.7 to 4.7 among the genotypes. In addition, during the ripening period, some fruits lost their commercial value. In another research paper, Gruz et al. [44] presented the average fruit weight in ripe stage (between 191 and 206 DAFB) as 8.51 ± 0.26 g and 8.62 ± 0.83 g, concluding that fruit weight increase is slow and gradual during the natural ripening process.

Although Sebek et al. [6] indicated low variability between samples in terms of fruit weight, fruit width, fruit length, and petiole length based on genotypes, they observed an interesting aspect: The fruit weight of “Royal medlar” cultivar is three times greater than the fruit weight of medlar genotype “Pomoravka” (seedless variety from Bijelo Polje, Montenegro). A different conclusion from Haciseferogullari et al. [20], who assumed that changes in physical properties of fruits about the same size were probably due to environmental conditions.

## 3. Chemical Composition

Baird and Thieret [5] reviewed the medlar from almost all points of view, starting “from antiquity”. They wrote about the origin (geographical, etymology, and existence history) of medlar, its chemical composition, morphology, and utilization. Edwards et al. [64], in a review on the chemistry of the *Crataegus* genus, mentioned the determination of total soluble sugars and phenolic acids in medlar fruits. Two years later, Acosta-Estrada et al. [65] also mentioned medlar in a review, emphasizing the bound phenolics in ripe medlar fruit. From various papers used in this review, the major components (as general composition) of medlar are summarized in Table 5.

The presented composition varies with a series of factors, such as the following: the cultivar/genotype, region of cultivation, and the degree of fruit maturity and ripeness. Among the reviewed research papers, several studies presented the chemical composition of *Mespilus germanica* L. fruits depending on several factors, and their relevant findings are presented in the following section.

The mineral composition of *Mespilus germanica* L. fruits, in terms of macro-elements and microelements, was analyzed by several authors (Table 6). By using inductively coupled plasma atomic emission spectrometer (ICP-AES), Haciseferogullari et al. [20] determined the mineral content of medlar fruit. The highest concentration was obtained for K (8052.91 mg/kg), followed by S, Ca, B, and P. Moreover, traces of Cr, Ti, and V were determined. In previous studies, Glew et al. [43] analyzed a series of minerals (Al, Ba, Ca, Cu, Co, Fe, K, Li, Mg, Mn, Na, Ni, P, Sr, Ti, and Zn) of medlar fruit and showed their high quantities of K (7370 µg/g dry wt), Ca (1780 µg/g dry wt), P (1080 µg/g dry wt), Mg (1661 µg/g dry wt), and Na (183 µg/g dry wt). The same researchers reported that the ripe medlar fruit is an important source of minerals and trace elements for the diet of populations in Western Asia (Turkey and Iran). They found significant differences in the levels of nutrients in medlar fruit related to different maturity stages [43]. In accordance with the studies of [20,43], Rop et al. [1] found that, at the ripe stage of medlar fruit of all the determined mineral compounds, the content of potassium was the highest (average 8320 ± 93 mg kg^−1^). Furthermore, they found some differences such as the following: a nine-fold higher accumulation of Mn, 3.5 times the amount of Ca, and 2.7 times that of P, 3.5-fold lower value for Fe. Accordingly, as previously mentioned, all of these differences in mineral composition can be caused by different growth, climate, and soil conditions or cultivation technique. Moreover, the ripening process has an influence on mineral composition, which tends to decrease these elements.

The results from several research papers indicate that medlar fruits usually contain minor amounts of fatty acids (Table 7). These are considered important precursors (the fatty acid path produces esters and C6 compounds via lipoxygenase) for various odorous volatile compounds (e.g., benzaldehyde, pentadecane, and tetradecane) and contribute to characteristic aroma, flavor, and nutritional value of the fruit during ripening [35,36,39,42]. Thus, even if the content of fatty acids varies with genotype/cultivar, palmitic acid (C 16:0), linoleic acid (C 18:2n-6), linolenic acid (C 18:3n-3), oleic acid (C 18:1n-9), stearic acid (C 18:0), arachidic acid (C 20:0), and behenic acid (C 22:0) are the most predominant fatty acids [35,36,39,42] during development and senescence processes. Among these acids, the highest percentage was obtained for palmitic acid. Although Ayaz et al. [35] found capric acid (C 10:0) and tridecanoic acid (C 13:0) in all ripening stages (between 39–154 DAFB), Canbay et al. [39], Glew et al. [42], and Ayaz et al. [35] did not detect them. The authors [35,36,39,42] reported that the most important changes in fatty acids’ composition of medlar fruit take place during medlar pulp softening. Glew et al. [42] considered that much of the potential benefit of fatty acids (C 18:2n-6 and C 18:3n-3) will be lost if the fruits are consumed 3–4 weeks after harvest. This idea confirms the findings of Ayaz et al. [35]: The level of linoleic acid and α-linolenic acid from the ripe hard fruits (60.0 and 13.5% of dry wt) decreased throughout ripening to a low of 28.7 and 5.6% of dry wt. Contrary to this, Ayaz et al. [35] emphasized a sudden increase in the content of some minor unsaturated fatty acids (palmitoleic acid, vaccenic acid, and erucic acid) at 187 DAF in the ripest, fully softened, and darkened pulp of medlar.

In addition to the determinations made, Canbay et al. [39] explained these major changes in the fatty acids’ composition of medlar fruits as the following: During fruit ripening and senescence, cell disorganization is accompanied by enzymatic disruption of lipoproteins membranes resulting in variation in lipid composition. They also assumed that decreasing chemical components in fruits during the ripening process could be explained in two ways: the involvement of ethylene in the ripening (first stage of senescence) and senescence process or the involvement of degradative lipolytic enzymes that metabolize endogenous lipids in senescing membranes.

Several authors analyzed the influence of different ripening stages on the content of other chemical compound (Table 8), such as the following: carbohydrates [38,40,42,62,66], organic acids [1,2,3,32,38,40,41,42,49,62,66], proteins [38,40], aldehydes [48,49], alcohols [49], esters [49], and terpenes [49]. As mentioned previously, following harvest period, medlar fruits can have a relatively short shelf life during which they undergo profound changes in texture, color, and flavour. Rop et al. [1] observed statistically significant decreases for ascorbic acid during fruit softening (except between stages 134 DAFB and 144 DAFB). This decrease in ascorbic acid was emphasized by Aydin and Kadioglu [38] too.

In the case of soluble proteins, Aydin and Kadioglu [38] observed that after a decrease during development, these compounds increased during ripening probably because of the ripening and senescence enzymes. This occurred for carbohydrates as well [38]; the level of glucose continuously increased during the development and ripening of medlar. This explains why the unripe medlar fruit has an astringent taste (high level of pro-anthocyanidin and low sugar content).

Most of the volatile components of fruits are mainly formed by esters, alcohols, acids, aldehydes, ketones, lactones, terpenoids, or apocarotenoids. These volatile aroma compounds appear during the ripening process through different metabolic pathways [49]. Among these constituents, organic acids are of increasing interest because of their role in the most important metabolic pathways of carbohydrates, lipids, and proteins [40]. Thus, several authors (Table 8) identified and quantified the main organic acids in fruits such as the following: ascorbic acid, citric acid, malic acid, oxalic acid, tartaric acid, fumaric acid, succinic acid, quinic acid, hexanoic acid, dodecanoic acid, tetradecanoic acid, pentadecanoic acid, and hexadecenoic acid. The data obtained in their studies confirm that medlar fruits represent a rich source of organic acids; their organic acid content per 100 g was greater than usual human daily consumption [40].

Selcuk et al. [66] indicated that malic acid was the most abundant organic acid, followed by succinic, quinic, oxalic, and citric acids in medlars, even in storage conditions. In general, a gradual decrease in malic acid content was observed during the entire storage period for both 1 MCP (1-Methylcyclopropene) treated fruit and control fruits. The fruit treated with 1-MCP also maintained high citric acid levels during storage, and this is probably due to the delay in the ripening process that results in decreasing organic acids levels.

Pourmortazavi et al. [48] and Velickovic et al. [49] studied the volatile compounds from medlar seeds by using supercritical fluid extraction followed by GC-MS analysis and from medlar fruits by using GC-MS analysis respectively. From medlar seeds, only three components were identified in the volatile oil: benzaldehyde, pentadecane, and tetradecane, the first one being the major component. In that study, the authors compared the supercritical fluid extraction method with hydro distillation and found an interesting result: supercritical fluid extraction products were markedly different from the corresponding hydrodistilled oil. Moreover, the authors considered that the supercritical fluid extraction method offers important advantages over hydro distillation (shorter extraction time, cost, and cleaner features) and contributes to the automation of the pharmaceutical industry [48]. On the other hand, Velickovic et al. [49] determined the changes in the volatile composition of medlar fruits during their two ripening stages: unripe and fully ripe stage. They found that the chemical compounds were aldehydes, alcohols, esters, acids, and terpenes, and C-6 aldehydes and alcohols were quantitatively dominant, among them.

Phenolic compounds represent a special and diverse class of plant secondary metabolites. Although they are known to be non-nutrient compounds, phenolics are reported to have multiple influences: tissue maturation processes, defense mechanisms, and sensory qualities of plant-derived food products (astringency, bitterness, and aroma) [44]. Several authors analyzed different medlar plant parts for antioxidant compounds (phenolics, flavonoids, carotenoids, etc.) and antioxidant capacity (Table 9, Table 10 and Table 11). The interest in phenolic acids comes from their potential protective role against oxidative damage, inflammation, cancer, cardiovascular diseases, and stroke. Researchers have found that phenolic compounds have strong antioxidant properties. Phenolic compounds are thought to contribute to the health effects of plant-derived products by scavenging free radical species, inhibiting free radical formation, and preventing oxidative damage to DNA [45].

The main conclusion from the presented data is that the concentrations of phenolic compounds and antioxidative capacity are significantly influenced by the stage of medlar fruit maturation and genotype. Moreover, an important decrease in total phenolic compounds occurs during ripening stages of medlar fruits [1]. For example, at 134 DAFB (ripening phase), the total phenolics content was 170 ± 1 mg gallic acid equivalent for 100 g fresh matter, but at the 174 DAFB stage, the content of phenolics was of 54% of that value. This decrease in phenolic compounds is closely related to the increasing polyphenol oxidase activity [1]. During the last two ripening stages (193 and 214 DAFB), the phenolic compounds decreased no matter what solvent for extraction was used (acetone, methanol, ethanol 80% or water) [34]. Another interesting idea presented by Rop et al. [1] is that antioxidants operate through different pathways; one method alone is not sufficient for evaluating the antioxidant activity of fruits and does not represent the entire antioxidant capacity of pure compounds.

Due to the fact that polyphenols are reducing agents, they can react with Folin–Ciocalteu reagent exactly as vitamin C, vitamin E, and carotenoids do. Consequently, this determination method is considered to be inappropriate for the total phenolics content determination (Folin–Ciocalteu reagent reacts with several non-phenolic reducing compounds—organic acids, sugars, and amino acids). In this case, the results will include higher phenolic compound values than in reality [1].

Among phenolic compounds, several authors determined p-aminobenzoic acid, caffeic acid, chlorogenic acid, p-coumaric acid, gallic acid, quercetin, protocatechuic acid, rutin, and vanillin as major phenolic compounds and catechin, epicatechin, ferulic acid, quercitrin, and resveratrol as minor phenolic compounds [3,45]. The values obtained for these compounds are influenced by the genotype. Moreover, flavonoids (a class of polyphenolic compounds) act as antioxidants, antimicrobials, photoreceptors, visual attractants, feeding repellents, and light screening substances in plants [62]. Rop et al. [1] observed that during fruit maturation, quercetin and its glycosylated derivates (glucosides and rhamnosides), were the most abundant flavonols. They consider that the sensory qualities of medlar fruit are extremely complicated, and vanillin is considered an aroma quality parameter for these fruits. Resveratrol was also identified, and it is known as a in vivo strong antioxidant [3].

Another group of compounds with known antioxidant activity by scavenging oxygen radicals and reducing oxidative stress in the organism include carotenoids. They possess preventive activity against a wide range of diseases (cardiovascular disease, hepatic fibrogenesis, solar light induced erythema, human papillomavirus persistence, and some cancer types) [47]. For the extraction of carotenoids, several authors recommended a wide range of solvent mixtures such as the following: methanol/tetrahydrofuran (THF) (50:50 *v*/*v*), ethyl acetate (100%), ethanol/hexane, acetone/ethanol/hexane, ethyl acetate/hexane, or acetone/hexane.

## 4. Storage Conditions for Medlar

Generally speaking, the medlar fruit is a typical climacteric one, meaning that it reaches full consuming maturity in a few days after harvest. Medlar fruit is very perishable and susceptible to skin and flesh browning, fast softening, and water loss after harvest. The results of these postharvest processes include the decrease in its edible and commercial value [66]. In order to avoid fast softening and browning during postharvest handling and storage and to increase the shelf life of this fruit, several authors have tried to find methods to accomplish these aims by using a Palliflex storage system and 1-methylcyclopropene treatment [66]; Palliflex storage system with low O_2_ and CO_2_ atmosphere [62]; 28-homobrassinolide [63]; or modified atmosphere packaging and methyl jasmonate [67].

Palliflex storage system is used for short-term or long-term storage under specific conditions (the desired O_2_ and CO_2_ concentrations can be set for each individual pallet). It is also known that 1-methylcyclopropene inhibits ethylene, which facilitates softening and senescence of fruits. The results emphasized that the firmness values of all the variants decreased with storage time and the used dose of 1-MCP. Thus, in control and 0.2 µL/L 1-MCP treated fruit, the process was more pronounced than 0.4 and 0.6 µL/L 1-MCP treated fruit. The retention of firmness is very important for long term storage of medlars [66]. In another research paper, the same authors analyzed the influence of Palliflex storage system and modified atmosphere packaging on physiological properties, qualities, and storage period for some medlar cultivar [62]. The results showed that, for all the treatment variants, the contents of total phenolic, total flavonoid, total condensed tannin, ascorbic acid, antioxidant activity, and organic acids decreased during storage, while no significant changes were detected in the content of sugars. It was also shown that the softening and skin browning slowed.

Another experiment for increasing the postharvest life of medlars was made by Ekinci et al. [63]. They determined the effects of postharvest brassinosteroid treatment on the storage quality of medlar fruit and emphasized the influence of 28-homobrassinolide applications on the physical and chemical properties of medlar fruit stored for 60 days. Their conclusion was that treating medlar fruits with 5 μM 28-homobrassinolide after harvest retained higher quality over a longer period [63].

Ozturk et al. [67] analyzed the influence of modified atmosphere packaging and methyl–jasmonate on the quality and health promoting properties of medlar fruit during the storage period. The addition of methyl–jasmonate to the modified atmosphere packaging (already known to have a good influence in preserving the medlar fruits quality) was also found to be effective in slowing down the reduction in ascorbic acid (vitamin C), one of the most important vitamins for human nutrition.

## 5. Molecular Biology Analyses

There are only a few research articles regarding molecular biology analyses on *Mespilus germanica* L. These papers focus on the analyses of relationship between *Mespilus* and *Crataegus* genus or on analyses that emphasize the polymorphism between the apparent different *Mespilus germanica* L. genotypes/cultivars worldwide. Lo et al. [29] analyzed, in their research paper, the fact that *Mespilus* and *Crataegus* are two distinct genera and the relationship between *M. canescens* and other *Mespilus* or *Crataegus* taxa. They used ITS (Internal Transcribed Spacers) and LEAFY (intron2 of the floral homeotic gene), representative for the nuclear genome, and also trnS-trnG, psbA-trnH, trnH-rpl2, and rpl20-rps12—four non-coding (intergenic) chloroplast regions. Their research revealed that *Mespilus* comprises not only *Mespilus germanica* species (from Eurasia) but also *Mespilus canescens* (from USA). They concluded that molecular and morphological data indicate no clear genetic distinction between *Crataegus* and *Mespilus.* The best taxonomic solution (based on both the molecular phylogeny and the morphological data) is to include the genus *Mespilus* in *Crataegus* as a new monotypic section. This does not interfere with the actual nomenclature (see also [29]).

Schaefer et al. [30] made some analysis regarding the genetic diversity of medlar germplasm (10 *M. germanica* and 1 *M. canescens* samples) using microsatellite markers: 21 apple SSR (Simple Sequence Repeat) primer pairs and 2 pear SSR primer pairs, previously reported to be useful in the tribe *Pyreae*. They observed that SSRs from apples were successfully able to distinguish most of the accessions medlar samples. Moreover, they sustained the idea of diverse genetic backgrounds represented in the medlar samples collection and the necessity of additional SSRs in order to confirm genetic identity and relationships in all accessions in the medlar collection.

Another group of researchers, Zarei et al. [33], performed phylogenetic analysis among samples from fruit trees of the *Rosaceae* family by using RAPD (Random Amplified Polymorphic DNA) markers. It is well known that RAPD markers have been used to analyze genetic diversity, construction of genetic maps, population structures, phylogeny studies of supposed related species and genera, etc. In their analyses, all primers used in the experiments were highly polymorphic, producing 85 clear and reproducible bands. Even if these authors used another type of primer in their experiments, the results were similar to those obtained by Schaefer et al. [30] with microsatellite markers. Thus, *Mespilus* and *Crataegus* have the highest genetic similarity among the studied samples. At the same time, they have higher similarity with respect to members of *Pyrus* compared to the *Malus* genus. Moreover, different species from *Crataegus* were clearly separated and grouped together, and the *Mespilus* genus had some common genetic similarities with three other genera (in their study) and might represent the branching point for the development of different pome fruit trees.

The most recent study on phylogenetic position of *Mespilus* was conducted by Liu et al. [26]. Their study analyzed a high number of samples (131 chloroplast genomes representing 115 species from 31 genera). They concluded that three species of *Amelanchier* (from W North America), one species of *A. ovalis* (from Europe), and two species of *A. sinica* and *A. asiatica* (E Asia) form a strong clade that is sister to *Malacomeles*. At the same time, eight *Amelanchier* species (from E North America) formed a clade with *Peraphyllum*. These two major clades are sister to each other and are, together, sister to the *Crataegus-Mespilus–Hesperomeles* clade [26].

## 6. Biological Activities of *Mespilus germanica* L.

### 6.1. Antioxidant Properties

Several research papers provided valuable information on the antioxidant capacity of medlar plant parts (fruits, leaves, bud flowers, or stem bark). Antioxidants (phenolics and flavonoids) from fruits and vegetables have been associated with the decrease in incidences of heart disease, some cancers, or age-related degenerative diseases. Medlar plants were shown to be a forgotten rich source of polyphenolic and antioxidant compounds. Table 12 summarizes the main findings regarding the antioxidant potential of *Mespilus germanica* L., as well as the responsible classes of compounds (as presented by the authors).

Due to the fact that one method alone cannot be utilized to completely evaluate antioxidant activity, different antioxidant capacity tests with different approaches and mechanisms have been carried out [1,3,10,39,41,44,45,46,55]. Gulcin et al. [45] demonstrated the antioxidant and radical scavenging mechanism of LEM (lyophilized extract of medlar) by using different in vitro bioanalytical methodologies: DPPH free radical scavenging, DMPD+ scavenging, total antioxidant activity (ferric thiocyanate method), reducing power using two methods (Fe^3+^-Fe^2+^ transformation and Cuprac assays), superoxide anion radical scavenging generated, hydrogen peroxide scavenging, and metal chelating on ferrous ions (Fe^3+^) activities. They found that LEM possessed powerful Fe^3+^ reducing abilities with a Trolox equivalent (0.69 μg TE) (Table 12). Moreover, Rop et al. [1] presented the connection between the decrease in phenolic content and total antioxidant activity. Antioxidant activity measured using the ABTS test on medlar cultivars varied based on ascorbic acid equivalents from 100 to 180 AAE. 

Unlike other authors, Nabavi et al. [46] and Isbilir et al. [55] studied the antioxidant capacity of different medlar plant parts and not only fruits but also leaves, stem bark, and flower bud. They found [46] that the radical-scavenging activities of all the extracts (methanol or water extracts) increased with increasing concentration. Thus, WB (water extract–bark stem) with the highest phenol content showed the highest activity (IC_50_ = 10.7 ± 0.6 μg·ml^−1^), which is comparable with vitamin C and quercetin. There were no significant differences between stem bark and leaf extracts (aqueous and methanol) in terms of reducing power. Moreover, the fruit methanol extract exhibited better activity than other extracts (IC_50_ = 247 ± 12.2 μg·ml^−1^). The main conclusion of their research was that stem bark extract (both aqueous and methanol) showed the most activity in nearly all tests.

Isbilir et al. [55] found that the leaves and flower bud extracts had good free radical scavenging activity at the highest concentrations. The DPPH scavenging activities of leaf extract were determined to be 41.3 ± 0.7% and 63.4 ± 2% at the concentrations of 100 and 250 µg/mL, respectively. According to the results of the DPPH scavenging method, IC_50_ values were determined to be 157 μg/mL for leaf, 260 μg/mL for bud flower and 695 μg/mL for fruits. They concluded that due to total phenolic and flavonoid contents, DPPH radical scavenging and β-carotene bleaching activities of medlar plant parts were determined to be in the following order: leaf > flower bud > fruit [55].

Similar results were obtained by Ercisli et al. [41], who found that the determination of antioxidant activities by β-carotene–linoleic acid and 2-diphenyl-1-picryhydrazyl (DPPH) free radical scavenging assays resulted in an average 80.8 % and 46.6 μg/mL fresh weight DPPH, respectively.

On the other hand, Akbulut et al. [3] considered the genotype to influence the extent of antioxidant activity in medlar fruits at a statistically significant level (*p* < 0.05). Total antioxidant activity was the highest in genotype KRD-6 (187 mg AAE per 100 g fresh fruit sample) and lowest in genotype KRD-12 (124 mg AAE per 100 g fresh fruit).

As a general remark, it can be observed that most authors assign antioxidant potential to the total phenolic and total flavonoids content. They all consider medlar to be a valuable source of antioxidant compounds.

### 6.2. Antimicrobial Activity

Medicinal plants, especially the endemic and edible plants in certain locales, are particularly important for the development of new drugs due to their ability to produce compounds with antioxidant and antimicrobial activities and their importance in human health [10]. Thus, *Mespilus germanica* L. is a medicinal plant with therapeutic effects historically [5]. Despite the medical benefits and significant therapeutic effects of medlar, there are only a few scientific papers about the antimicrobial properties of this medicinal plant [10]. In this context, several authors evaluated the antibacterial effects of different extracts of medlar against microorganisms from various environments in last decade [10,46,50,51,52,57] (Table 13). Thus, Niu et al. [52] analyzed the in vitro antibacterial effect of two medlar extracts (water extract and ethanol extract) on pathogenic bacteria *Staphylococcus aureus* and *Klebsiella pneumonia*. Their results showed that the medlar extract was moderately sensitive to *Staphylococcus aureus*, and its inhibiting effect on *Klebsiella pneumoniae* was particularly significant. In addition, the antibacterial effect of ethanol extract was greater than water extract.

Similar results were obtained by Ahmady-Asbchin et al. [50] who evaluated the antibacterial effects of methanolic and ethanolic medlar leaf extract against bacteria isolated from hospital environments (*Pseudomonas aeruginosa*, *Staphylococcus aureus*, and *Escherichia coli*). The results showed that the methanolic extract of medlar leaf (instead of ethanolic extract as previously studied) inhibited the growth of all *Pseudomonas aeruginosa* and *Escherichia coli* strains (except one) and four strains of *Staphylococcus aureus*. Moreover, the minimum inhibitory concentration (MIC) for all the strains was 125 mg/mL.

Davoodi et al. [51] evaluated the antibacterial activity of hydro-acetonic extract of medlar leaf against *Klebsiella pneumoniae*, *Vibrio cholera*, *Escherichia coli*, and *Shigella dysenteriea*. The extract showed best inhibitory (MIC = 3.333 ± 0.0233) and bactericidal (minimal bactericidal concentration (MBC) = 5.833 ± 0.065) activities against *Klebsiella pneumoniae*. The lowest MIC was observed against *Vibrio cholera* (6.667 ± 0.048), and the lowest MBC was observed against *E. coli* and *Shigella dysenteriea* (9.167 ± 0.042).

Safari et al. [10] evaluated different standard (ATCC) bacterial strains that include Staphylococcus aureus, Staphylococcus epidermidis, Streptococcus pneumoniae, Enterococcus faecalis, Salmonella typhi, Salmonella paratyphi, Escherichia coli, Klebsiella pneumoniae, Pseudomonas aeruginosa, Yersinia enterocolitica, Serratia marcescens, Shigella dysenteriae, and Citrobacter freundii. They tested different concentrations of methanolic extract of medlar leaves in order to emphasize antibacterial activity against both Gram-positive and Gram-negative bacteria. Higher inhibition activity was observed against S. aureus (one of the most common causes of several diseases and responsible for food poisoning). The experiments showed interesting results, meaning that the methanolic extracts of medlar leaves emphasized relatively higher antibacterial activity against Gram-positive than against Gram-negative bacteria. Moreover, the antibacterial effect of this extract against S. aureus, S. epidermis, and E. coli was stronger than that of gentamicin [10].

In previous studies, there are several mentions about two antibiotics produced by the medlar plant [8,14]. In 1964, two antibiotic cyclopentoid monoterpenes were isolated and identified as genipic acid and genipinic acid (its carbomethoxyl derivative). Another group of researchers tested the effect of ethanolic extract of medlar on cutaneous leishmaniasis [57]. This group of infectious diseases is caused by species of the genus *Leishmania* and is a significant cause of morbidity and mortality in several countries. At present, *Leishmania* affects 6 million people in 98 countries. Due to the fact that there is no effective anti-leishmania cure, the researchers attempted to find new plant constituents as the source of new chemotherapeutic compounds. As previously mentioned, plants are rich in a wide variety of secondary metabolites (tannins, terpenoids, alkaloids, and flavonoids) and are found to have in vitro antimicrobial properties [10,46,50,51,52]. Since this severe disease has a long treatment period, used for over 55 years with parental drug administration and several toxic side effects (pentavalent antimonials), it was necessary to find alternative solutions [57]. Thus, the use of ethanolic *Mespilus germanica* L. extracts in laboratory experiments reduces both lesion size and the number of parasites. During treatment, 40% concentration (leaves ethanolic extract) had the maximum effect on cured scar diameters (compared to 60% and 80% variants). The authors suggested that these ethanolic extracts had potential for topical wound healing, representing motivation for further exploration of anti-leishmania agents.

## 7. Usage of Medlar

As a medicinal plant, forgotten, neglected, and abandoned *Mespilus germanica* L. represents a suitable source of a wide range of secondary (and primary) metabolites: essential oils, antimicrobials, vitamins, antioxidants, minerals, etc. Based on some reports from World Health Organization, almost 80% of the world’s people use traditional medicine for their primary health care needs [4]. Medicinal plants have several advantages: fewer side effects, effectiveness, and relatively low-cost production. The most common uses of medlar plants and the articles that we analyzed for this paper are presented in Table 14. The diversity of recipes with medlar is amazing, especially in countries with a tradition of the cultivation or presence of medlar plants.

## 8. Conclusions

*Mespilus germanica* L. represents a forgotten and abandoned species of fruit tree that is becoming more and more interesting and attractive due to the special properties of its fruits. The current study aimed to present a complete picture of the currently known morphology, composition, biological properties, usage, and storage conditions for medlar.

It is used (fruits, leaves, bark, and bud flowers) in traditional medicine in a variety of diseases or medical conditions, as well as in gastronomic areas, and in a wide range of recipes (traditional/local recipes).

The chemical composition of *Mespilus germanica* L. fruits, leaves, bark, or bud flowers revealed high concentrations in antioxidant compounds (polyphenols and flavonoids), carotenoids, vitamins, minerals, etc. Highlighting the composition and properties of the medlar fruits is a very important aspect in order to rediscover this valuable fruit tree and to stimulate its cultivation and consumption.

The literature study revealed a lack of information (only few related studies exist) on molecular biology analysis for identifying the polymorphism between cultivars from different countries and for identifying different genes that encode for special properties. Moreover, although medlar trees are present in many places than is presented in research papers, no information (scientific literature) from other countries was found.

Future research directions should include, as the industrial perspective, the possibility of using the biocompounds from *Mespilus germanica* L. in the pharmacology industry or food industry. The content in microelements, polyphenols, and vitamins render these fruits excellent raw materials for obtaining natural bioproducts that are standardized, with a role in maintaining the health of the human body. Moreover, an important advantage of this fruit tree is the period of ripening in fruits—late autumn—which renders it an important source of fruits for the winter (food supply when other fruits are missing from the market). Regarding the valorification of medlar fruits, it should be used in small entrepreneurial business development.

## Figures and Tables

**Table 1:** Different names of medlar worldwide.

Country—Language	Name	References
Azerbaijan	Ezgil	[6]
Armenian	Zkereni	[7]
Chinese	Ou Cha	[8]
Czech	Mišpule obecná	
Danish	Mispel	
Dutch	Mispel; Mispelboom	
Eastonian	Harilik astelpihlakas	
Finnish	Mispeli	
French	Merlier, Néfle Commune (Fruit), Néflier,Néflier Commun (Tree)	
Georgia	Bushmala	[6]
	Mushmala, Zghmartli	[7]
German	Aschperln, Asperl, Deutsche Mispel, Dürgen, Dürrlitzen, Dörrlitzen, Echte Mispel, Hespelein, Hundsärsch, Mispel, Mispelbaum, Mispelche, Nespoli, Nispel	[8]
Greek	Mespilea E Germaniki	[8]
	Mousmoulo (fruit), Mousmoulia (tree)	[3]
Hungarian	Naspolya	[8]
Iran	Kondos	[8]
	Kounos	[4]
	Azgil	[9]
	Conos, Condos	[10]
Italian	Nespola, Nespolo, Nespolo volgare	[8]
Japanese	Seiyou Karin	
Latin	Mespilum	[11]
Polish	Nieszpułka zwyczajna	[8]
Portuguese	Nêsperas, Nespereira, Nespereira (Tree), Nespereira-Da-Europa	
Romanian	Mașmule, Mișculă, Mostachiu, Născale, Hospurușe, Scoruțe nemțești	[12]
	Hascul	[13]
	Gorun, Mișcul, Mostoc, Scoruș nemțesc	[14]
	Moșmon, Măcieș, Moșmol, Mostochin	[15]
	Nuspui	[16]
Russian	Mushmula, Mushmula Obyknovennaia	[8]
Slovenian	Navadna nešplja	
Spanish	Níspero (Tree), Níspero Común, Níspero Europeo, Nísperoeuropeo, Níspola (Fruit), Nispolero	
Swedish	Mispel, Tysk mispel	
Turkish	Mumula, Mušmula	[3,8]
	Döngel, Beşbıyık	[6]
Ukrainian	Mushmula	[8]

**Table 2:** Physico-chemical characteristics of medlar leaves.

Plant/Genotype	Leaf Length (cm)	Leaf Width (cm)	Leaf Stalk Length (mm)	Leaf Area (cm^2^)	Reference
Healthy mature plants	6.5–10.0	2.9–3.5	0.44–0.81	No data	[53]
C1	9.7 ± 0.3	3.96 ± 0.10	No data	29.76 ± 1.22	[23]
N1	12.18 ± 0.26	5.60 ± 0.16		48.8 ± 1.84	
M1	11.24 ± 0.20	3.94 ± 0.11		32.17± 1.35	
M2	10.7 ± 0.32	4.9 ± 0.15		36.77 ± 2.07	
M3	11.02 ± 0.21	4.42 ± 0.10		35.56 ± 1.21	
T1	8.8 ± 0.16	3.5 ± 0.08		22.95 ± 0.72	
E1	9.92 ± 0.24	4.13 ± 0.08		29.31 ± 1.11	
Cr1	9.20 ± 0.15	3.89 ± 0.11		25.40 ± 0.85	
Cr2	9.56 ± 0.21	3.97 ± 0.07		27.45 ± 1.01	

Where: healthy mature plants—from Kocaeli province, Turkey; M1, M2, and M3 (Mătăsari; Southwestern Romania); Cr1 and Cr2 (Croici; Southwestern Romania); N1 (Nanov; South Romania); C1 (Craiova; Southwestern Romania); T1 (Turnu-Ruieni; West Romania); E1 (Ezeriș; West Romania).

**Table 3:** Morphological characteristics of medlar fruits.

Days after Full Bloom (DAFB)	Harvest Date	Fruit Skin and Pulp Color	State of Ripeness	Reference
172	26 october 2000	ripe, skin partly dark brown, fruit table soften, pulp whitish, and partly brownish	Mature, ripe	[35]
187	10 November 2000	very ripe, skin and pulp fully dark brown, and fruit soften	Ripe	
191	15 November 2003	skin completely brown, pulp white, and fruit half soft	Ripe	[44]
206	30 November 2003	skin completely dark brown; pulp whitish–brownish (50%–50%); fruit soft and juicy	Ripe	
193	18 November 2003	skin brownish, pulp white, and fruit hard	Mature, ripe	[34]
207	2 December 2003	skin completely brown; pulp white–partly brownish (60%–40%) around core; fruit half soft	Ripe	
164	21 November 2008	the skin was becoming brown, and the pulp was mostly white; estimated as consumption maturity when fruits become edible	Mature, ripe	[1]
174	1 December 2008	the skin and the pulp were completely brown and soft	Ripe	

**Table 4:** Variability of the main morphometric characteristics of *Mespilus germanica* L. plant parts (fruits and stone) according to the authors from different countries.

Stage/Year	Fruit Weight (g)	Fruit Diameter (mm)	Fruit Length (mm)	pH	Stone Weight (g)	Stone Width (mm)	Stone Length (mm)	Reference
39 DAFB/1999	2.9 ± 0.1	0.7 ± 0.1	No data	No data	No data	No data	No data	[36]
66 DAFB/1999	5.1 ± 0.1	1.4 ± 0.2	No data	No data	No data	No data	No data	
102 DAFB/1999	6.6 ± 0.1	2 ± 0.1	No data	No data	No data	No data	No data	
131 DAFB/1999	8.8 ± 0.1	2.5 ± 0.2	No data	No data	No data	No data	No data	
154 DAFB/1999	7.5 ± 0.2	2.9 ± 0.2	No data	No data	No data	No data	No data	
Different stages of ripening/1999	No data	1.8–2.5 (cm)	No data	No data	No data	No data	No data	[38]
191 DAFB/2003	8.51 ± 0.26	No data	No data	No data	No data	No data	No data	[44]
206 DAFB/2003	8.62 ± 0.83	No data	No data	No data	No data	No data	No data	
Maturity stage/2003	12.0 ± 0.2	27.7 ± 0.2	31.4 ± 0.2	4.3 ± 0.2	No data	No data	No data	[20]
Harvest stage/2008	21.6	32.3	32.2	No data	No data	No data	No data	[39]
Harvest stage/2009	18.6	31.2	31.9	No data	No data	No data	No data	
Commercial maturation stage/2011	11.21–16.42	28.44–36.62	27.45–38.88	No data	No data	No data	No data	[41]
Harvest stage/2010–2012	No data	No data	No data	3.4–3.86	No data	No data	No data	[6]
Harvest stage/2010–2012	21.4–25.5	31.5–36.2	34.5–38.4	No data	No data	No data	No data	
Maturity stage/2011–2012	17.71–32.46	21.07–41.05	18.25–38.27	3.54–3.92	No data	No data	No data	[32]
Maturity stage/2011–2012	15.99–37.54	17.49–43.63	14.96–35.68	3.54–3.99	No data	No data	No data	
Consuming stage/2011–2012	No data	No data	No data	3.75–3.98	No data	No data	No data	
Consuming stage/2011–2012	No data	No data	No data	3.76–4.00	No data	No data	No data	
No stage data/2012	38.36	4.22 (cm)	4.34 (cm)	4.26	3.21	7.9	11.43	[24]
Physiological maturity/2012	20.21 ± 0.13	30.37 ± 0.26	31.76 ± 0.22	4.01 ± 0.035	No data	No data	No data	[17]
Ripening period/2012	15.48 ± 0.14	26.34 ± 0.31	28.30 ± 0.18	4.70 ± 0.037	No data	No data	No data	
Commercial maturation stage/2013	12.3–23.6	No data	No data	No data	No data	No data	No data	[3]
Harvest time/2013–2014	5.2–20.1	21.2–33.3	21.0–33.6	3.68–4.02	0.16–0.45	6.4–9.0	10.4–12.5	[53]
Storage conditions/2015	No data	No data	No data	3.24–3.70	No data	No data	No data	[62]
Maturity stage/2018	No data	21.00 ± 9.70	27.00 ± 4.50	No data	No data	5.80 ± 0.16	8.30 ± 0.64	[2]
Commercial maturity stage/2018	24.14	35.11	34.30	No data	No data	No data	No data	[19]
Storage conditions/2019	No data	No data	No data	3.87–4.52	No data	No data	No data	[63]

**Table 5:** Major chemical components of medlar fruits.

Category	Compound	Reference
Acids	Citric acid, Dodecanoic acid, Fumaric Acid, Hexadecanoic acid, Hexanoic acid, Malic acid, Oxalic acid, Quinic acid, Pentadecanoic acid, Succinic acid, Tartaric acid, Tetradecanoic acid	[39,41,48,63,64]
Aldehyde	Benzaldehyde, Benzene acetaldehyde, (*E*,*Z*)-2,4-Decadienal, (*E*,*E*)-2,4-Decadienal Hexanal, (*E*)-2-Decenal, Furfural, (*E*)-2-Hexenal, *n*-Nonanal, (*Z*)-2-Nonen-1-al	[48,49]
Alcohols	Hexanol, (*Z*)-3-Hexenol, Phenyl ethyl alcohol	[49]
Carbohydrates	Fructose,Glucose, Hexose, Pentose, Sucrose	[37,41,63,64]
Carotenoids	β-carotene, Lycopene	[10,47]
Esters	Ethyl-hexadecanoate, Ethyl-octadecanoate (18:0), Ethyl-octadecenoate (18:1), Ethyl-oleate	[49]
Fatty acids	Arachidic acid, Behenic acid, Capric acid, Cerotic acid, cis-11-Eicosenoic acid, cis-11,14-Eicosadienoic acid, Erucic acid, Lauric acid, Lignoceric acid, Linoleic acid, α-Linolenic acid, Linolelaidic acid, Margaric acid, Myristic acid, Myristoleic acid, Oleic acid, Palmitic acid, Palmitoleic acid, Pentadecanoic acid, Phthalic acid, Stearic acid, Tridecanoic acid, Vaccenic acid	[8,34,35,38,41]
Total flavonoids	Total flavonoids, Quercetin	[9,44,45,54,63,64]
Minerals	Al, As, B, Ba, Ca, Cd, Co, Cr, Cu, Fe, In, K, Li, Mg, Mn, Mo, Na, Ni, P, Pb, S, Se, Sr, Ti, V, Zn	[1,8,40,19,23,42]
Proteins	Proteins	[38,41]
Total phenols	Caffeic acid, p-Coumaric acid, Ellagic acid, Ferulic acid, Pyrogallol, Total phenols	[3,9,31,40,44,45,54,63,64]
Terpenes	*p*-Cymen-8-ol, *p*-Cymene, γ-Eudesmol, *α*-Murolene, Phellandrene, Terpinen-4-ol, *α*-Terpinene, *γ*-Terpinene, Terpinolene, *α*-Terpineol	[49]
Vitamins	Vitamin C	[2,3,32,38,40,41,42,62,66]
	α-Tocopherol	[45,55]
Others	Pentadecane, Tetradecane	[48]

**Table 6:** Mineral composition of *Mespilus germanica* L.

Region of Cultivation	Minerals	Observation	Method	Reference
	**mg kg^−1^ Dry Matter**			
Turkey (Trabzon)	Al = 10.1 ± l.2; Ba = 19.7 ± 0.4; Ca = 1780 ± 3.2; Co < 0.1; Cu = 3.6 ± 0.2; Fe = 13.4 ± 1.2; K = 7370 ± 67; Li = 0.02 ± 0.01; Mg = 66 ± 8.1; Mn = 10.2 ± 0.1; Na = 183 ± 5.4; Ni = 0.3 ± 0.1; P = 1080 ± 12; Sr = 16.3 ± 0.3; Ti = 0.5 ± 0.1; Zn = 7.l ± 0.4	Ripe stage (October 1999)	ICP-AES	[43]
Turkey (Egirdir—Isparta)	Al = 44.0 ± 1.3; B = 356.5 ± 17.6; Ca = 883.1 ± 21.5; Cr = 1.4 ± 0.0; Fe = 91.9 ± 1.6; In = 1.6 ± 0.1; K = 8052.9 ± 12.3; P = 344.8 ± 6.4; Pb = 2.2 ± 0.5; S = 3544.8 ± 13.4; Se = 6.6 ± 0.7; Ti = 1.9 ± 0.1; V = 0.6 ± 0.1; Zn = 4.0 ± 0.5	Ripe stage (November 2003)	ICP-AES	[20]
Iran (Province of Mazandaran)	Ca = 25,359 ± 0.10; Cr = 1.82 ± 0.14; Fe = 164.53 ± 1.04; K = 7751.63 ± 1.87; Mg = 787.69 ± 0.86; Na = 649 ± 0.54; Zn = 41.13 ± 0.00	November 2008	ICP-AES	[9]
Czech Republic	Ca = 2754 ± 86; Fe = 27.52 ± 2.20; K = 8725 ± 92; Mg = 913 ± 50; Na = 124 ± 12; P = 961 ± 41; Zn = 5.90 ± 0.39	164 DAFB (21.11.2008)	atomic absorption spectrometry	[1]
Czech Republic	Ca = 2695 ± 115; Fe = 27.60 ± 1.45; K = 8320 ± 93; Mg = 842 ± 41; Na = 121 ± 16; P = 938 ± 32; Zn = 6.10 ± 0.50	174 DAFB (1.12.2008)	atomic absorption spectrometry	[1]
Turkey (Anatolia)	Al = 4.515; As = 0.068; B = 7.959; Ca = 1186.378; Cd = 0.018; Cr = 0.241; Cu = 0.496; K = 6962.6441; Fe = 5.983; Li = 0.301; Mg = 1070.08; Na = 82.800; Ni = 0.593; P = 763.425; Pb = 0.133; S = 131.238; Sr = 5.802; V = 3.200; Zn = 1.087	Ripe stage (2012)	ICP-AES	[24]
	**mg/100g Fresh Mass**			
Turkey (Coruh valley)	Ca = 73; Fe = 7.2; K = 792; Mg = 55; P = 39; Mn = 0.5; Zn = 0.5	Commercial maturity stage	atomic absorption spectrometry	[41]

**Table 7:** Fatty acids composition of medlar fruits.

Fatty Acid	Value	Stage of Ripeness	Method	Reference
Capric acid (C10:0)	n.d. (mg/g dry wt)	Ripe stage *	GC	[42]
	6.7 ± 0.4 (µg/g dry wt)	154 DAFB, ripe stage	GC	[36]
	n.d. (%)	187 DAFB, ripe stage	GC	[35]
	n.d. (%)	No data	GC-MS	[39]
Lauric acid (C12:0)	0.37 (%)	Ripe stage	GC-MS	[9]
	2.6 ± 0.1 (mg/g dry wt)	Ripe stage *	GC	[42]
	6.9 ± 2.9 (µg/g dry wt)	154 DAFB, ripe stage	GC	[36]
	1.4 ± 0.40 (%)	187 DAFB, ripe stage	GC	[35]
	0.80 ± 0.11 (%)	No data	GC-MS	[39]
Tridecanoic acid (C13:0)	n.d (mg/g dry wt)	Ripe stage *	GC	[42]
	7.7 ± 2.2 (µg/g dry wt)	154 DAFB, ripe stage	GC	[36]
	n.d. (%)	187 DAFB, ripe stage	GC	[35]
	n.d. (%)	No data	GC-MS	[39]
Myristic acid (C14:0)	0.38 (%)	Ripe stage	GC-MS	[9]
	2.3 ± 0.3 (mg/g dry wt)	Ripe stage *	GC	[42]
	9.6 ± 0.4 (µg/g dry wt)	154 DAFB, ripe stage	GC	[36]
	1.1 ± 0.17 (%)	187 DAFB, ripe stage	GC	[35]
	1.50 ± 0.02 (%)	No data	GC-MS	[39]
Myristoleic acid (C14:1)	2.4 ± 0.3 (mg/g dry wt)	Ripe stage *	GC	[42]
	7.4 ± 1.9 (µg/g dry wt)	154 DAFB, ripe stage	GC	[36]
	n.d. (%)	187 DAFB, ripe stage	GC	[35]
	0.30 ± 0.09 (%)	No data	GC-MS	[39]
Pentadecanoic acid (C15:0)	1.6 ± 0.1 (mg/g dry wt)	Ripe stage *	GC	[42]
	3.6 ± 0.1 (µg/g dry wt)	154 DAFB, ripe stage	GC	[36]
	0.9 ± 0.11 (%)	187 DAFB, ripe stage	GC	[35]
	0.10 ± 0.01 (%)	No data	GC-MS	[39]
Palmitic acid (C16:0)	6.97 (%)	Ripe stage	GC-MS	[9]
	70.4 ± 0.8 (mg/g dry wt)	Ripe stage *	GC	[42]
	420 ± 9.6 (µg/g dry wt)	154 DAFB, ripe stage	GC	[36]
	36.9 ± 1.13 (%)	187 DAFB, ripe stage	GC	[35]
	35.35 ± 1.20 (%)	No data	GC-MS	[39]
Palmitoleic acid (C16:1)	0.49 (%)	Ripe stage	GC-MS	[9]
	1.4 ± 0.3 (mg/g dry wt)	Ripe stage *	GC	[42]
	8.9 ± 0.2 (µg/g dry wt)	154 DAFB, ripe stage	GC	[36]
	0.6 ± 0.03 (%)	187 DAFB, ripe stage	GC	[35]
	0.30 ± 0.03 (%)	No data	GC-MS	[39]
Stearic acid (C18:0)	1.78 (%)	Ripe stage	GC-MS	[9]
	15.7 ± 0.7 (mg/g dry wt)	Ripe stage *	GC	[42]
	68.0 ± 3.1 (µg/g dry wt)	154 DAFB, ripe stage	GC	[36]
	7.9 ± 1.19 (%)	187 DAFB, ripe stage	GC	[35]
	8.53 ± 0.25 (%)	No data	GC-MS	[39]
Oleic acid (C18:1n-9)	11.45 (%)	Ripe stage	GC-MS	[9]
	6.7 ± 0.2 (mg/g dry wt)	Ripe stage *	GC	[42]
	250.6 ± 1.7 (µg/g dry wt)	154 DAFB, ripe stage	GC	[36]
	3.5 ± 0.03 (%)	187 DAFB, ripe stage	GC	[35]
	4.35 ± 0.37 (%)	No data	GC-MS	[39]
Vaccenic acid (C18:1n-7)	2.9 ± 0.2 (mg/g dry wt)	Ripe stage *	GC	[42]
	24.1 ± 0.6 (µg/g dry wt)	154 DAFB, ripe stage	GC	[36]
	1.5 ± 0.01 (%)	187 DAFB, ripe stage	GC	[35]
	0.85 ± 0.11 (%)	No data	GC-MS	[39]
Linoleic acid (C18:2n-6)	0.22 (%)	Ripe stage	GC-MS	[9]
	55 ± 1.5 (mg/g dry wt)	Ripe stage *	GC	[42]
	1291.7 ± 7.7 (µg/g dry wt)	154 DAFB, ripe stage	GC	[36]
	28.7 ± 1.65 (%)	187 DAFB, ripe stage	GC	[35]
	29.10 ± 1.70 (%)	No data	GC-MS	[39]
α-Linolenic acid (C18:3n-3)	10.8 ± 0.4 (mg/g dry wt)	Ripe stage *	GC	[42]
	359.9 ± 3.2 (µg/g dry wt)	154 DAFB, ripe stage	GC	[36]
	5.6 ± 0.38 (%)	187 DAFB, ripe stage	GC	[35]
	4.93 ± 0.79 (%)	No data	GC-MS	[39]
Arachidic acid (C20:0)	2.99 (%)	Ripe stage	GC-MS	[9]
	8 ± 0.3 (mg/g dry wt)	Ripe stage *	GC	[42]
	36.6 ± 1.05 (µg/g dry wt)	154 DAFB, ripe stage	GC	[36]
	4.2 ± 0.23 (%)	187 DAFB, ripe stage	GC	[35]
	3.20 ± 0.85 (%)	No data	GC-MS	[39]
cis-11-Eicosenoic acid (C20:1n-9)	0.4 ± 0.1 (mg/g dry wt)	Ripe stage *	GC	[42]
	4.2 ± 0.2 (µg/g dry wt)	154 DAFB, ripe stage	GC	[36]
	0.2 ± 0.11 (%)	187 DAFB, ripe stage	GC	[35]
	0.12 ± 0.08 (%)	No data	GC-MS	[39]
cis-11,14-Eicosadienoic acid (C20:2n-6)	0.4 ± 0.0 (mg/g dry wt)	Ripe stage *	GC	[42]
	0.2 ± 0.15 (%)	187 DAFB, ripe stage	GC	[35]
	0.11 ± 0.01 (%)	No data	GC-MS	[39]
Behenic acid (C22:0)	2.45 (%)	Ripe stage	GC-MS	[9]
	8.3 ± 0.4 (mg/g dry wt)	Ripe stage *	GC	[42]
	39.7 ± 1 (µg/g dry wt)	154 DAFB, ripe stage	GC	[36]
	4.4 ± 0.83 (%)	187 DAFB, ripe stage	GC	[35]
	4.00 ± 0.75 (%)	No data	GC-MS	[39]
Erucic acid (C22:1n-9)	1.3 ± 0.1 (mg/g dry wt)	Ripe stage *	GC	[42]
	3.3 ± 0.0 (µg/g dry wt)	154 DAFB, ripe stage	GC	[36]
	0.7 ± 0.18 (%)	187 DAFB, ripe stage	GC	[35]
	0.50 ± 0.03 (%)	No data	GC-MS	[39]
Lignoceric acid (C24:0)	2.47 (%)	Ripe stage	GC-MS	[9]
	3.3 ± 0.5 (mg/g dry wt)	Ripe stage *	GC	[42]
	24.6 ± 0.6 (µg/g dry wt)	154 DAFB, ripe stage	GC	[36]
	2.1 ± 0.23 (%)	187 DAFB, ripe stage	GC	[35]
	2.50 ± 0.25 (%)	No data	GC-MS	[39]
Margaric acid (C17:0)	0.21 (%)	No data	GC-MS	[39]
Linolelaidic acid (C18:2, n-6,9)	24.01 (%)	No data	GC-MS	[39]
Cerotic acid (C26:0)	0.26 (%)	No data	GC-MS	[39]

Where n.d. = not determined (depend on the ripening stage, not on the lack of determination); * = 3 weeks after harvest.

**Table 8:** Other chemical compounds in medlar fruits.

Compound	Values	Ripe Stage/Plant Part	Method	Reference
**Carbohydrates**				
Fructose	mg 100 g^−^^1^ fw			
	2153.1 ± 4.7	1 WAH	ethanolic extract	[42]
	2230.8 ± 0.4	2WAH		
	117.5 ± 1.7	3WAH		
	22.7 ± 1.3	4WAH		
	7948–8033	Commercial maturity stage	No data	[66]
	7336–7851	Commercial maturity stage	No data	[62]
Glucose	mg g^−1^ dry wt			
	0.55–9.99	Different stages of fruit ripening	phenol-sulphuric acid method	[38]
	mg 100 g^−1^ fw			
	734.8 ± 3.6	1 WAH	ethanolic extract	[42]
	845.2 ± 1.9	2 WAH		
	548.3 ± 0.6	3 WAH		
	16.9 ± 1.4	4 WAH		
	6095–6891	Commercial maturity stage	No data	[66]
	5669–6137	Commercial maturity stage	No data	[62]
Hexose	mg g^−1^ dry wt 143.1–510.9	Different stages of fuit ripening	phenol-sulphuric acid method	[38]
Pentose	mg g^−1^ dry wt 189.6–662.1	Different stages of fuit ripening	phenol-sulphuric acid method	[38]
Sucrose	mg 100 g^−1^ fw			[42]
	228.4 ± 4.4	1 WAH	ethanolic extract	
	145.3 ± 2.3	2WAH		
	18.6 ± 1.1	3WAH		
	1.4 ± 0.1	4WAH		
**Acids**				
Ascorbic acid	mg g^−1^ dry wt			
	3.3–6.7	Different stages of fuit ripening	procedure of Shieh and Sweet	[38]
	mg 100 g^−1^ fw			
	9.0 ± 0.8	1 WAH	ethanolic extract	[42]
	5.6 ± 0.5	2WAH		
	2.8 ± 0.2	3WAH		
	No data	4WAH		
	0.7	Ripened fruits	HPLC	[40]
	59 ± 2 17 ± 1	134 DAFB 174 DAFB	HPLC-ED	[1]
	8.00–30.00	Maturity stage	No data	[32]
	6.40–36.67	Consuming stage	No data	[32]
	11.3–14.4	Commercial maturity stage	reflectometry	[41]
	0.78–12.1	Commercial maturity stage	No data	[66]
	1.37–12.10	Commercial maturity stage	No data	[62]
	mg % dry matter 90.30 ± 0.73	Fruit	No data	[2]
	mg/100 g fw 13–24	Commercial maturity stage	No data	[3]
Citric acid	mg 100 g^−1^ fw			
	420.2 ± 1.0	1 WAH	ethanolic extract	[42]
	250.8 ± 1.3	2WAH		
	71.4 ± 1.5	3WAH		
	0.3 ± 0.0	4WAH		
	16.41	Ripened fruits	HPLC	[40]
	3.6–22.96	Commercial maturity stage	No data	[66]
	2.94–21.71	Commercial maturity stage	No data	[62]
Malic acid	mg 100 g^−1^ fw			
	434 ± 1.3	1 WAH	ethanolic extract	[42]
	572.9 ± 0.9	2WAH		
	307.5 ± 0.8	3WAH		
	1 ± 0.1	4WAH		
	415.08	Ripened fruits	HPLC	[40]
	1273–1919	Commercial maturity stage	No data	[66]
	1185–1733	Commercial maturity stage	No data	[62]
Oxalic acid	mg 100 g^−^^1^ fw			
	54.73	Ripened fruits	HPLC	[40]
	25.29–45.62	Commercial maturity stage	No data	[66]
	26.37–35.29	Commercial maturity stage	No data	[62]
Tartaric acid	mg 100 g^−^^1^ 111.57	Ripened fruits	HPLC	[40]
Fumaric acid	mg 100 g^−^^1^ 0.79	Ripened fruits	HPLC	[40]
Succinic acid	mg 100 g^−^^1^ fw			
	452.9–596.9	Commercial maturity stage	No data	[66]
	424.5–570.0	Commercial maturity stage	No data	[62]
Quinic acid	mg 100 g^−^^1^ fw			
	573.7–789.86	Commercial maturity stage	No data	[66]
	337.94–534.65	Commercial maturity stage	No data	[62]
	%	Medlar fruits, unripe	GC-MS	[49]
Hexanoic acid	tr.			
Dodecanoic acid	tr.			
Tetradecanoic acid	tr.			
Pentadecanoic acid	tr.			
Hexadecanoic acid	6.13			
	%	Medlar fruits, ripe	GC-MS	[49]
Hexanoic acid	5.44			
Dodecanoic acid	tr.			
Tetradecanoic acid	0.09			
Pentadecanoic acid	0.12			
Hexadecanoic acid	8.87			
**Proteins**				
Soluble protein	mg g^−1^ dry wt 0.17–0.61	Different stages of fuit ripening	method of Bradford	[38]
Crude protein	% 3.3–4.3	Commercial maturity stage	Kjeldahl method	[41]
**Aldehydes**				
	%	Medlar seeds	SFE	[48]
Benzaldehyde	98.49			
Pentadecane	1.08			
Tetradecane	0.43			
	%	Medlar fruits, unripe	GC-MS	[49]
Hexanal	32.81			
Furfural (*E*)-2-Hexenal	0.12			
Benzaldehyde	43.47			
Benzene acetaldehyde	tr.			
*n*-Nonanal	tr.			
(*Z*)-2-Nonen-1-al	tr.			
(*E*)-2-Decenal	tr.			
(*E*,*Z*)-2,4-Decadienal	tr.			
(*E*,*E*)-2,4-Decadienal	tr.			
	%	Medlar fruits, ripe	GC-MS	[49]
Hexanal	6.53			
Furfural	2.12			
(*E*)-2-Hexenal	tr.			
Benzaldehyde	0.40			
Benzene acetaldehyde	0.28			
*n*-Nonanal	0.27			
(*Z*)-2-Nonen-1-al	0.99			
(*E*)-2-Decenal	0.20			
(*E*,*Z*)-2,4-Decadienal	0.10			
(*E*,*E*)-2,4-Decadienal	0.63			
**Alcohols**				
	%	Medlar fruits, unripe	GC-MS	[49]
(*Z*)-3-Hexenol	2.27			
Hexanol	12.12			
Phenyl ethyl alcohol	tr.			
	%	Medlar fruits, ripe	GC-MS	[49]
(*Z*)-3-Hexenol	9.47			
Hexanol	42.57			
Phenyl ethyl alcohol	0.45			
**Esters**				
	%	Medlar fruits, unripe	GC-MS	[49]
Ethyl-hexadecanoate	tr.			
Ethyl-oleate	tr.			
Ethyl-octadecenoate (18:1)	tr.			
Ethyl-octadecanoate (18:0)	tr.			
	%	Medlar fruits, ripe	GC-MS	[49]
Ethyl-hexadecanoate	0.35			
Ethyl-oleate	0.11			
Ethyl-octadecenoate (18:1)	tr.			
Ethyl-octadecanoate (18:0)	tr.			
**Terpenes**				
	%	Medlar fruits, unripe	GC-MS	[49]
*α*-Terpinene	tr.			
*p*-Cymene	0.11			
Phellandrene	0.37			
*γ*-Terpinene	tr.			
Terpinen-4-ol	0.18			
γ-Eudesmol	0.11			
Terpinolene	tr.			
*p*-Cymen-8-ol	tr.			
*α*-Terpineol	tr.			
*α*-Murolene	tr.			
	%	Medlar fruits, ripe	GC-MS	[49]
*α*-Terpinene	2.86			
*p*-Cymene	tr.			
Phellandrene				
*γ*-Terpinene	1.02			
Terpinen-4-ol	12.56			
γ-Eudesmol	0.15			
Terpinolene	tr.			
*p*-Cymen-8-ol	tr.			
*α*-Terpineol	tr.			
*α*-Murolene	tr.			

Where: WAH = weeks after harvest; tr. = traces.

**Table 9:** Antioxidant compounds of *Mespilus germanica* L. fruits.

Responsible Compound	Value	Ripening Stage	Reference
**Phenols (Total)**			
Total phenolics as GAE	mg 100 g^−1^ fm		
	117 ± 1	164 DAFB	[1]
	920.51 ± 51.59	Maximum in maturity stage	[32]
	453.09 ± 23.33	Maximum in consuming stage	
	122.55–985.03	Commercial maturity stage	[66]
	86.4–763.03		[62]
	157–227		[3]
	114–244		[41]
	mg g^−1^		
	25.08	No data	[45]
	7.26 ± 0.4	Commercial maturity stage	[41]
	16.5 ± 3.53	Fresh	[55]
**Flavonoids**			
Total flavonoids as QE	mg g^−1^		
	14.08 ± 1.1 Water extract	No data	[46]
	14.88 ± 1.2 Methanol extract	No data	
	1.99 ± 0.02	Fresh	[55]
	µg/g		
	2.39		[45]
	mg 100 g^−^^1^ fw	Commercial maturity stage	[66]
	73.32–1085.65		
	43.98–630.98	Commercial maturity stage	[62]
Other antioxidants			
	mg/kg	No data	[45]
Caffeic acid	4.9		
Ferulic acid	2.4		
Ellagic acid	0.2		
Quercetin	2.4		
a-Tocopherol	13.4		
Pyrogallol	3.6		
p-Coumaric acid	2.4		
Ascorbic acid	184.6		
	mg/100 g	No data	
Lycopene	nd		[47]
β-carotene	0.9 ± 0.0		
Lycopene	nd		
β-carotene	1.0 ± 0.0		

Where: GAE = gallic acid equivalent; QE = quercetin equivalent; nd = not determined.

**Table 10:** Antioxidant compounds of *Mespilus germanica* L. leaves.

Responsible Compound	Value	Ripening Stage	Reference
**Phenols**			
Total phenolics as GAE	mg g^−1^		
	60.3 ± 1.69	Fresh	[55]
	380.58 ± 0.73 Methanolic extracts	No data	[10]
**Flavonoids**			
Total flavonoids as QE	mg/g 14.77 ± 1.15	Fresh	[55]
	mg/g dray wt 75.169 ± 0.04	No data	[10]
**Other antioxidants**			
Carotenoids	µg/mL 3.43 ± 0.13	No data	[10]

Where: GAE = gallic acid equivalent; QE = quercetin equivalent.

**Table 11:** Antioxidant compounds of *Mespilus germanica* L. flower buds.

Responsible Compound	Value	Ripening Stage	Reference
**Phenols**			
Total phenolics as GAE	mg g^−1^ 50.3 ± 0.51	Fresh	[55]
**Flavonoids**			
Total flavonoids as QE	mg/g 6.54 ± 0.08	Fresh	[55]

Where: GAE = gallic acid equivalent; QE = quercetin equivalent.

**Table 12:** Antioxidant properties of different extracts obtained from *Mespilus germanica* L.

Plant Part	Extraction Method	Antioxidant Assay	Antioxidant Potential	Responsible Compounds	Reference
**Fruits**					
LEM fruits	Water extraction followed by lyophilization	DPPH, DMPD+ and O_2_—radical scavenging, Fe^2+^ chelating, Fe^3+^-Fe^2+^ reducing ability, Cu^2+^-Cu^+^ reducing ability, FRAP reducing ability	DPPH· scavenging: 0.62 μg TE	Total phenolics and flavonoids	[45]
			DMPD+ scavenging: 0.81 μg TE		
			O_2_-scavenging: 1.41 μg TE		
			Fe^2+^ chelating: 2.76 μg TE		
			Fe^3+^-Fe^2+^ reducing: 0.69 μg TE		
			Cu^2+^-Cu^+^ reducing: 0.43 μg TE		
			FRAP: 0.36 μg TE		
Fruits	hydrochloric acid:methanol:ACS water, in the ratio 2:80:18 (*v/v*)	inactivation of the cation ABTS^+^	100–180 mg AAE/100 g FM (different ripening stages)	Total phenolics	[1]
	n.m.	inactivation of the cation ABTS^+^	1.1 ± 0.2 mmol Trolox equivalents/L	n.m.	[39]
	80% ethanol	modified DPPH scavenging assay	15–95% (different ripening stages)	Total phenolics	[44]
	Methanol or water room temperature extraction	DPPH; Fe^3+^ reduction; Fe^2+^ chelating; nitric oxide-scavenging activity; scavenging of hydrogen peroxide	IC_50_ μg/ml	Total phenolics and flavonoids	[46]
			DPPH—419 ± 3.2/492 ± 33.1		
			Nitric oxide scavenging—247 ± 12.2/1328 ± 57.4		
			H_2_O_2_ scavenging activity—1138 ± 77.1/2333 ± 87.9		
			Fe^2+^ chelating ability—23.0/31.7 (methanol/water)		
	Methanol extraction	β-carotene bleaching; DPPH	IC_50_ μg/mL fresh weight	Total phenolics	[41]
			DPPH—46.6 (average)		
			β-carotene bleaching—80.8%		
	hydrochloric acid:methanol:ACS water, in the ratio 2:80:18 (*v/v*)	inactivation of the cation ABTS^+^	mg AAE/100 g fresh fruit 124–187	Total phenolics	[3]
	Ethanol extraction	DPPH; β-carotene bleaching	IC_50_ μg/mL DPPH—695 β-carotene bleaching—n.m.	Total phenolics and flavonoids	[55]
**Leaves**					
Leaves	Methanol or water room temperature extraction	DPPH; Fe^3+^ reduction; Fe^2+^ chelating; nitric oxide-scavenging activity; scavenging of hydrogen peroxide	IC_50_ μg/ml	Total phenolics and flavonoids	[46]
			DPPH—19.4 ± 1.3/19.8 ± 1.3		
			Nitric oxide scavenging—1129 ± 78.6/280.3 ± 16.8		
			H_2_O_2_ scavenging activity—58.1 ± 2.3/171 ± 14.1		
			Fe^2+^ chelating ability—24.6/30.1 (methanol/water)		
	Ethanol extraction	DPPH; β-carotene bleaching	IC_50_ μg/ml	Total phenolics and flavonoids	[55]
			DPPH—157		
			β-carotene bleaching—400		
	95% Methanol extraction	DPPH	69.43 ± 0.36%	Total phenolics and flavonoids	[10]
**Other plant parts**					
Stem bark	Methanol or water room temperature extraction	DPPH; Fe^3+^ reduction; Fe^2+^ chelating; nitric oxide-scavenging activity; scavenging of hydrogen peroxide	IC_50_ μg/ml	Total phenolics and flavonoids	[46]
			DPPH—11.4 ± 0.8/10.7 ± 0.6		
			Nitric oxide scavenging—376 ± 16.5/557.7 ± 25.1		
			H_2_O_2_ scavenging activity—427 ± 35.1/537 ± 23.6		
			Fe^2+^ chelating ability—28.4/504 ± 34.5 (methanol/water)		
Bud flowers	Ethanol extraction	DPPH; β-carotene bleaching	IC_50_ μg/ml	Total phenolics and flavonoids	[55]
			DPPH—260		
			β-carotene bleaching—960		

Where AAE = ascorbic acid equivalents; ABTS^+^ = 2,2′-azinobis(3-ethylbenzothiazoline-6-sulphonate); ACS = American Chemical Society; DMPD^+^ = N,N-dimethyl-p-phenylenediamine; DPPH = 1,1-diphenyl-2-picryl-hydrazil radical; FM = fresh matter; FRAP = ferric reducing ability of plasma; IC_50_ = half maximal inhibitory concentration; LEM fruits = lyophilized extract of medlar fruits; n.m. = not mentioned; TE = Trolox equivalent.

**Table 13:** Antimicrobial effects of *Mespilus germanica* L. extracts.

Plant Part	Extract Type/Bioassay	Test Against	Results		Reference
Leaves	methanolic extract/ agar disc diffusion method		MIC (mg/mL)		[10]
		*Staphylococcus aureus*	62.5		
		*Staphylococcus epidermidis*	62.5		
		*Salmonella typhi*	125		
		*Salmonella paratyphi*	62.5		
		*Escherichia coli*	125		
		*Klebsiella pneumoniae*	125		
		*Pseudomonas aeruginosa*	125		
		*Streptococcus pyogenes*	62.5		
		*Enterococcus faecalis*	125		
		*Yersinia enterocolitica*	62.5		
		*Serratia marcescens*	125		
		*Shigella dysenteriae*	125		
		*Citrobacter freundii*	125		
Leaves	methanolic extract/ agar disc diffusion method		MIC (mg/mL)		[50]
		*Pseudomonas aeruginosa*	125		
		*Staphylococcus aureus*	63–125		
		*Escherichia coli*	63–250		
	ethanolic extract/ agar disc diffusion method		MIC (mg/mL)		
		*Pseudomonas aeruginosa*	0–250		
		*Staphylococcus aureus*	0–500		
		*Escherichia coli*	0–500		
Leaves	ethanolic extract/ ethanolic extract in vaseline base rubbed topically	*Leishmania major*	- lesion diameters were remarkable reduced in treatment with concentrations 40% and 60% ethanolic extract compared to control group; - size of the lesions that received 80% concentration of ethanolic extracts had no statistically significant difference with control group		[57]
Leaves	70% acetone extract/ agar disc diffusion method		MIC (mg/mL)	MBC (mg/mL)	[51]
		*Klebsiella pneumoniae*	3.333 ± 0.0233	5.833 ± 0.065	
		*Vibrio cholera*	6.667 ± 0.048	lack of data	
		*Escherichia coli*	lack of data	9.167 ± 0.042	
		*Shigella dysenteriae*	lack of data	9.167 ± 0.042	
Fruits	water extract/ agar disc diffusion method		MIC (mg/mL)	MBC (mg/mL)	[52]
		*Staphylococcus aureus*	5	100	
		*Klebsiella pneumoniae*	2.5	50	
	50% ethanol extract/ agar disc diffusion method		MIC (mg/mL)	MBC (mg/mL)	
		*Staphylococcus aureus*	2.5	50	
		*Klebsiella pneumoniae*	0.625	2.5	

Where MIC = minimum inhibitory concentration value (mg/mL); MBC = minimal bactericidal concentration (mg/mL).

**Table 14:** Different possibilities of *Mespilus germanica* L. usage.

Usage Area	Usage	Plant Part	Reference
Traditional medicine “Folk medicine”	Hematopoietic	Leaves, fruits, bark	[4,57]
	Large intestine infection	Leaves, fruits, bark	
	Diarrhea	Leaves, fruits, bark	
	Internal hemorrhage	Leaves, fruits, bark	
	Cutaneous leishmaniasis -Sodden -Vaseline base applied topically	n.m. Leaves	
	Strengthen fine skin -Sodden	n.m.	
	Treatment intestinal inflammation - used in a little milk after removing skin and seeds	Fruits	[4]
	Elimination of throat abscess Gargle with sodden of leaves	Fruits	[57]
	Regurgitation disposal cholera - Sodden	Fruits	
	Stimulation treatment throat Sodden	Fruits	
	Strengthen nerves	Fruits	
	Elimination of stomach bloating	Fruits	
	Fattening	Fruits	
	Diuretic	Fruits, bark	
	Treatment of menstrual irregularities	Fruits	
	Fever disposal -Dry powder in alcohol (as washing the feet)	Bark	
	Enteritis	Pulp or syrup	[21]
	Diabetes	Leaves	
	Leaves decoction		[21]
	Leaves infusion		[55]
	Tuberculosis boiled and administered orally	Bark of the branches	[21]
	Abdominal pain	n.m.	
	Kidney and bladder of stones	n.m.	[1]
	Anti-influenza -Infusions, raw	Leaves, fruits	[56]
Gastronomy	Juice	Fruits	[4]
	Conserve	Fruits	
	Cooking jams	Fruits	
	Liqueur	Fruits	[6]
	Raw	Fruits	[1,4,56]
	Raw with cheese as a dessert	Fruits	[4]
	“Medlar cheese”	Fruits	
	Dessert -browning the fruit slices in butter and sprinkling them with cinnamon	Fruits	[59]
Poisson	-	Seed	[4]

Where n.m. = not mentioned.

## Data Availability

All data used for the review are included in the manuscript.
